# Fluctuation of the renal function after discharge from hospital and its effects on drug dosing in elderly patients – study protocol

**DOI:** 10.1186/s12882-015-0095-4

**Published:** 2015-07-07

**Authors:** Willemijn L. Eppenga, Wietske N. Wester, Hieronymus J. Derijks, Rein M.J. Hoedemakers, Michel Wensing, Peter A.G.M. De Smet, Rob J. Van Marum

**Affiliations:** Radboud University Medical Center, Radboud Institute for Health Sciences, IQ healthcare, Postbus 9101, 114, 6500 HB Nijmegen, The Netherlands; Department of Geriatric Medicine, Jeroen Bosch Hospital, ’s-Hertogenbosch, The Netherlands; ZANOB, ’s-Hertogenbosch, The Netherlands; Division of Pharmacoepidemiology and Clinical Pharmacology, Utrecht Institute of Pharmaceutical Sciences, Utrecht, The Netherlands; Laboratory of Clinical Chemistry and Haematology, Jeroen Bosch Hospital, ’s-Hertogenbosch, The Netherlands; Radboud University Medical Center, Department of Pharmacy, Nijmegen, The Netherlands; Department of General Practice and Elderly Care Medicine, VU University Medical Center Amsterdam, Amsterdam, The Netherlands

**Keywords:** Renal function, Elderly, Drug therapy management, Fluctuation, Study protocol

## Abstract

**Background:**

Chronic kidney disease (CKD) is associated with an increased mortality rate, risk of cardiovascular events and morbidity. Impaired renal function is common in elderly patients, and their glomerular filtration rate (GFR) should be taken into account when prescribing renally excreted drugs. In a hospital care setting the GFR may fluctuate substantially, so that the renal function group and therefore the recommended dose, can change within a few days. The magnitude and prevalence of the fluctuation of renal function in daily clinical practice and its potential effects on appropriateness of drug prescriptions after discharge from the hospital is unknown.

**Methods/design:**

This is a prospective observational study. Patients ≥ 70 years with renal impairment (eGFR <60 ml/min/1.73 m^2^) admitted to a geriatric ward are eligible to participate. Participants undergo blood sample collection to measure serum creatinine level at three time points: at discharge from hospital, 14 days, and 2 months after discharge. At these time points the actual medication of the participants is assessed and the number of incorrect prescriptions according to the Dutch guidelines in relation to their estimated renal function is measured. In addition, for a hypothetical selection of drugs, the need for drug dose adaptation in relation to renal function is measured. The outcome of interest is the percentage of patients that changes from renal function group after discharge from hospital compared to the renal function at discharge. In addition, the percentages of patients whose actual medications are incorrectly prescribed and for the hypothetical selection of drugs that would have required dose adaptation will be determined at discharge, 14 days and 2 months after discharge. For each outcome, risk factors which may lead to increased risk for fluctuation of renal function and/or incorrect drug prescribing will also be identified and analysed.

**Discussion:**

This study will provide data on changes in renal function in elderly patients after discharge from the hospital with a focus on the medications used. The benefits for healthcare professionals comprise of the creation, adjustment or confirmation of recommendations for the monitoring of the renal function after discharge from hospital of elderly patients.

## Background

Chronic kidney disease (CKD) is associated with an increased mortality rate, as well as with an increased risk of cardiovascular events and morbidity [1–3]. Because of the longer life expectancy, due to improved treatment of chronic diseases, the prevalence of impaired renal function increases [2–4]. In addition, there is a concern about prescribing drugs, which need dose adjustment in impaired renal function, especially in the elderly who are at risk for impaired renal function [4].

Up to one-third of the adverse drug reactions (ADRs) leading to hospital admission of elderly patients receiving outpatient care, may be related to impaired renal function [5, 6]. The ADRs were serious, because they resulted in or contributed to hospital admission. The problems with ADRs, including ADRs not leading to hospital admission, due to impaired renal function might be even greater in ambulatory care. Therefore, the consideration of renal function in ambulatory care drug therapy management (DTM) should be improved [5–7].

The estimated glomerular filtration rate (eGFR) is important in the clinical management of patients [8]. It is used for timely detection and management of declining renal function, to adjust the dosage of renally excreted drugs appropriately, and to avoid nephrotoxic drugs [1, 8, 9]. In the Netherlands, the eGFR is usually estimated in daily clinical practice with the Modification of Diet in Renal Disease (MDRD) formula. Dose adjustment of or refraining from renally excreted drugs commonly takes place categorically (see Table 1) [10].

**Table 1:** Renal function groups for drug dosing [10]

Group	Description	GFR (ml/min/1.73 m^2^)
1	Normal renal function	>80
2	Mild renal impairment	50–80
3	Moderate renal impairment	30–50
4	Severe renal impairment	<30
5	End stage renal disease (ESRD)	Requiring dialysis

In a hospital care setting a patient is closely observed and (repeated) creatinine measurements are easy to perform. Informal clinical observations suggest that the renal function may fluctuate so much that the renal function group and therefore the recommended dose, can change within a few days. A frequent scenario is that the patient is dehydrated at hospital admission with impaired renal function and during hospital admission the fluid balance is optimized and the eGFR increases.

The prevalence and the magnitude of fluctuations of the renal function after discharge from the hospital are still unknown. Since measurement of renal function is not routinely performed in the ambulatory care setting changes in renal function may remain unnoticed. It is possible that the eGFR may decrease, because of comprised fluid intake, or may increase because of the patient’s further recovery. Changes of renal function may lead to a shift to another renal function group for drug dosing (see Table 1) and thereby put patient at risk for not having optimal DTM. This may have unwanted clinical consequences, such as ADRs or insufficient effect. However, little is known about the actual prevalence of such scenarios in daily practice.

The aim of this study is to describe the changes in estimated renal function in elderly patients 14 days and 2 months after discharge from hospital compared to the value at discharge. Renal function will be classified according to the categories, which are defined in relation to DTM (see Table 1). In addition, incorrectly prescribed drugs in the actual medications of the patient according to the Dutch guidelines in relation to their renal function will be determined at discharge, 14 days and 2 months after discharge from hospital. Finally, the percentage of patients in whom the fluctuation would have required another dosage regimen, if they had been taking a hypothetical selection of drugs that require dose adaptation, will be determined. For each outcome, risk factors for the fluctuation of renal function and for incorrect prescribing will be examined.

## Methods/design

This study is conducted according to the Declaration of Helsinki. The study has been approved by the accredited Medical Ethical Committee of Brabant (formerly: METOPP). The Jeroen Bosch Hospital in ‘s-Hertogenbosch has provided local feasibility approval.

### Study designs and setting

This study is a prospective observational study and will be performed at the geriatric ward of the Jeroen Bosch hospital (JBZ), which is a teaching top-clinical hospital in The Netherlands serving 800 beds.

### Study population

All consecutive patients ≥ 70 years with renal impairment (eGFR <60 ml/min/1.73 m^2^ calculated with the MDRD formula (eGFR)) at admission to the geriatric ward will be asked to participate in the study. Furthermore, patients must remain community dwelling after discharge from hospital for at least 2 months. Exclusion criteria are patients with an eGFR < 10 ml/min/1.73 m^2^ and patients discharged for end-of-life care.

Patients eligible for the study will be invited and informed by their geriatrician. Informed consent is obtained by the researcher. In case the patient is incapacitated the patients’ legal guardian will be informed through written information and in case of participation the legal guardian will sign the informed consent. Permission to request the medication history at the community pharmacy will be asked at the same time. Patients can leave the study at any time for any reason without any consequences for their treatment.

### Study parameters

#### Primary study parameters

The main study parameter is the eGFR(MDRD). Serum creatinine level will be measured at 3 different time points, at discharge, 14 days and 2 months after discharge. The eGFR(MDRD) will automatically be calculated as follows:$175 \times {{\mathrm{S}}_{\mathrm{cr}}}^{\hbox{-} 1.154} \times \mathrm{ag}{\mathrm{e}}^{\hbox{-} 0.203} \times 1.212\ \left(\mathrm{if}\ \mathrm{black}\right) \times 0.742\ \left(\mathrm{if}\ \mathrm{female}\right)$ [11].

The estimated renal function will be classified according to the categories, which are commonly applied in relation to DTM (see Table 1). The main endpoint is the percentages of patients in whom the eGFR improves, deteriorates and remains unchanged within 2 months after discharge compared to their eGFR at discharge.

Improvement is defined as a change to a better renal function group as shown in Table 1 compared to the renal function group at discharge. Deterioration is defined as the change towards a worse renal function group as shown in Table 1 compared to the renal function group at discharge. Unchanged is defined as no change in renal function group.

#### Secondary study parameters

At each time point the recommended dose or contraindication for each drug the patient uses is determined in accordance with the eGFR of the patient and the Dutch guidelines for drug-dosing in chronic kidney disease [12]. A secondary endpoint will be the percentage of patients in whom the prescribed drugs are not in accordance with the Dutch guidelines at discharge, 14 days and 2 months after discharge from hospital.

Possible reasons for changes in the percentage of incorrect prescribed drugs at the different time points will be identified. Besides fluctuation of eGFR, other reasons could be starting a new drug not adjusted to renal function or discontinuation of a drug, which needed adjustment in renal impairment.

The average number of incorrectly prescribed drugs per patient and the type of drugs most often incorrectly prescribed at the three different time points will be determined.

The top 10 most frequently prescribed drugs in the elderly outpatients (>70 years) that require dose adaptation in patients with renal impairment are selected from the database of the Dutch foundation for pharmaceutical statistics (see Appendix 1) (Table 3). The recommended doses or contraindications of this hypothetical selection of drugs will be determined at each time point in relation to the renal function of the patient and subsequently compared with the recommended doses or contraindications at discharge. The endpoints will be the percentages of patients in whom a change in the medication would be needed at 14 days and at 2 months after discharge compared to the medication needed at discharge.

Potential risk factors at admission and during admission which may predict fluctuation between renal function groups will be determined. These risk factors are presented in Table 2.

**Table 2:** Data collection overview

	Time-points			
Parameter	At admission	At discharge	14 days after discharge	2 months after discharge
Serum creatinine level	√	√	√	√
Medication history		√	√	√
*Potential risk factors*				
Age	√			
Gender	√			
Weight	√			
Length	√			
Incapacitated patient	√			
Admission via emergency department or planned admission		√		
Reason for admission (diagnosis)		√		
C-reactive protein (CRP)	√			
Duration of admission		√		
Co-morbidities		√		
Nutritional status (SNAQ-score)	√			
Hydration status	√			
All serum creatinine values measured during admission		√		
Specific items in medication history				
• NSAID use 2 weeks prior to admission	√			
• Polypharmacy		√		
• Use and dose of diuretics		√		
• Use of NSAIDs		√		
• Use of RAS-inhibitors		√		
• Medications which influences creatinine production^a^		√		

^a^ These medications are: glucocorticosteroids, cimetidine, trimethoprim, fenofibrate (except gemfibrozil), calcitriol and alfacalcidol [22–24]

### Data collection

Table 2 shows the detailed data collection at each time-point.

When patients are admitted to the geriatric ward a blood sample is almost always routinely taken for a range of tests including serum creatinine value and C-reactive protein (CRP).

Prior to discharge (max. 2 days) a blood sample will be taken to measure serum creatinine. At discharge the patients are given two laboratory forms to collect blood samples 14 days and 2 months after discharge to measure creatinine. The patient will be reminded to go to the nearest general practitioners laboratory for blood sample collection 14 days and 2 months after discharge. Initially the blood samples were taken at the patient’s nearest general practitioners laboratory. This was changed to blood sample collection at home because patients often did not show up at the laboratory.

At admission the following variables are collected as part of usual care: age, gender, weight, length, use of NSAIDs in the two weeks prior to admission, nutritional status and hydration status. The nutritional status will be defined with the Simplified Nutritional Appetite Questionnaire (SNAQ) [13] and the hydration status will be observed and judged by the physician.

The following parameters will be collected retrospectively per patient: all creatinine values measured during admission, admission via emergency department or a planned admission, reason for admission (diagnosis), duration of admission, co-morbidities, and medication orders during admission.

After two months the researcher will obtain the medication history (from at least 6 months prior to discharge to 2 months after discharge) of the patient from the community pharmacy.

### Measurement of serum creatinine

All blood samples (heparinized plasma) will be analyzed at the Clinical Chemistry and Hematology department of JBZ. Serum creatinine levels will be measured in blood samples with the isotope dilution mass spectrometry (IDMS)-traceable Jaffe method on a Dimension Vista 1500 system (Siemens Healthcare Diagnostics) [14].

The results of the creatinine measurements will be reported to the general practitioner and/or the geriatrician as soon as the creatinine value is available. If necessary, adjustment of the medication can be made according to the Dutch guidelines by the general practitioner and/or the geriatrician. Reporting the creatinine value will not have an influence on the results. We will carefully examine the medication histories and identify changes made after reporting the creatinine value.

### Statistical power estimation

This study focuses on the fluctuation of renal function in elderly patients within two months after discharge from hospital. We are both interested in the percentage patients that change from one to another renal function group, but also in the direction of these changes (improvement or deterioration).

Raosoft sample size calculator (http://www.raosoft.com/samplesize.html) was used to estimate the number of subjects required to detect a change in renal function group in 15 % of the population with a 95 % confidence level and a 5 % margin of error. The recommended sample size is 195 subjects.

In order to detect a difference in percentage of patients in whom the change in renal function group improved or deteriorated the sample size is calculated based on the McNemar test (https://www.statstodo.com/SSizMcNemar_Pgm.phpPgm.php). Assuming that 5 % of the patients improve and 15 % of the patients deteriorate after discharge from hospital, power (1-beta) is 0.8 and probability of type I error (alpha) is 0.05, the sample size calculated is 155.

A sample size of 195 subjects should be sufficient for answering both questions in this study. Assuming that 20 % of the included patients are lost to follow-up, the number of patients needed to include is 195/0.8 = 244.

### Statistical analysis

For each patient data will be collected and archived in a validated data file. Errors and missing data will be monitored during data-collection, and complemented or corrected whenever possible. After completion of data-sampling, data will be checked for logical consistency (e.g., out of reach scores) and then finalised and locked. This file will be the basis for all further data analyses.

To analyse if the percentages of patients in which the renal function group improves, deteriorates and remains unchanged 14 days and 2 months after discharge from hospital compared to the renal function group at discharge are statistically significant, the McNemar-Bowker test will be used (categorical data with repeated measurements within one patient).

The secondary outcome is the percentage of patients in whom the prescribed drugs are not in accordance with the Dutch guidelines. To test the differences of the concordance of the medication to Dutch guidelines at the different time points, the McNemar test will be used (binary data with repeated measurements within one patient). The same test will be used to analyse the percentage of patients in whom a change in the medication would be needed if they had been using the top 10 most frequently prescribed drugs in elderly outpatients (>70 years) that require dose adaptation in patients with renal impairment.

In further explorative analyses, we use various analytical methods (e.g., logistic regression analysis and nominal logistic regression analysis) to identify potential determinants of outcomes.

## Discussion

DTM in patients with renal impairment is an important issue. Efforts are being taken to reduce drug therapy errors in these patients, for example by introducing clinical decision support systems [15, 16]. An educational intervention providing a list of frequently used drugs and their dosing schedule already reduced the number of drug dosing errors [17]. Special attention should be paid to older patients with renal insufficiency and polypharmacy who are using high risk medications such as anticoagulants (e.g., vitamin K antagonists, direct oral anticoagulants (DOAC)), diuretics, cardiovascular agents, analgesics, and anti-diabetic agents [18, 19]. All these studies and recently published guidelines for drug dosing in renal impairment reflect on our daily clinical practice in a hospital care setting. Every time when a new eGFR is reported the correct drug or drug dose is reviewed by the hospital pharmacist. Advices may change from one day to another.

Guidelines, such as KDIGO, do not offer advice about monitoring of the renal function after hospital discharge [20, 21]. In addition, it is tempting for community pharmacists and general practitioners to rely on the latest eGFR measured in the hospital for DTM after discharge. It is uncertain how stable the eGFR is in elderly patients who have recently been admitted to the hospital. This might be especially the case when eGFR changes were frequent during hospital admission. This study is designed to address the gap in monitoring the natural course of the renal function after discharge from the hospital with attention to the medications used in the elderly. If fluctuation of the eGFR is present at great extent this study will address whether closer monitoring of renal function and adaptation of renally excreted drugs after discharge will be needed. In addition, the benefits for healthcare professionals comprise of creation, adjustment or confirmation of recommendations for monitoring renal function after discharge from hospital of elderly patients.

## Appendix 1: Top 10 most frequently prescribed medications in elderly patients

**Table 3:** Top 10 most frequently prescribed drugs in the elderly outpatients (≥70 years) that require dose adaptation in patients with renal impairment^a^

	Drug	Dosing advice in renal impairment
1	furosemide	10–30 ml/min
		Starting dose as in normal renal function.
		If necessary, increase the dose guided by effect and indication.
		In case the effect is inadequate, replace furosemide by bumetanide.
2	metformin	30–50 ml/min
		Starting dose 2 × 500 mg metformin
		Then, increase the dose gradually to a standard maintenance dose.
		10–30 ml/min
		Contraindicated.
3	hydrochlorothiazide	10–30 ml/min
		Avoid hydrochlorothiazide
4	enalapril	30–50 ml/min
		Starting dose is 5 mg once daily.
		If necessary, increase the dose guided by clinical effect.
		If the prescriber is a general practitioner the maximum dose is 10 mg.
		If the prescriber is a specialized physician the dose may be higher.
		10–30 ml/min
		Starting dose is 2.5 mg once daily.
		If necessary, increase the dose guided by clinical effect.
		If the prescriber is a general practitioner the maximum dose is 5 mg.
		If the prescriber is a specialized physician the dose may be higher.
5	perindopril	30–50 ml/min
		If the prescriber is a general practitioner the maximum dose is 2 mg.
		If the prescriber is a specialized physician the dose may be higher.
		10–30 ml/min
		If the prescriber is a general practitioner the maximum dose is 2 mg every 48 h.
		If the prescriber is a specialized physician the dose may be higher.
6	digoxin	10–50 ml/min
		After digitalization, the starting dose is 0.125 mg once daily.
		Then, dose adjustment guided by clinical effect.
7	bumetanide	10–30 ml/min
		Starting dose as in normal renal function.
		If necessary, increase the dose to a maximum of 10 mg per day.
8	bisoprolol	10–30 ml/min
		Starting dose 50 % of the dose as in normal renal function
		If necessary, increase the dose to a maximum of 10 mg per day.
9	alendronic acid	10–30 ml/min
		Use is not recommended.
10	spironolacton	10–50 ml/min
		Monitor serum potassium levels regularly.

^a^ These prescription data were obtained from the Dutch “Foundation for Pharmaceutical Statistics (SFK)” in 2012

## Abbreviations

ADR(s): Adverse Drug Reaction(s)
CKD: Chronic Kidney Disease
CRP: C-Reactive Protein
DOAC: Direct Oral Anticoagulants
DTM: Drug Therapy Management
eGFR: estimated Glomerular Filtration Rate
GFR: Glomerular Filtration Rate
IDMS: Isotope Dilution Mass Spectrometry
JBZ: Jeroen Bosch Ziekenhuis
KDIGO: Kidney Disease Improving Global Outcomes
MDRD: Modification of Diet in Renal Disease
NSAID(s): Non-Steroidal Anti-Inflammatory Drug(s)
SNAQ: Simplified Nutritional Appetite Questionnaire
